# Quantifying cultural tightness-looseness in Ecuador

**DOI:** 10.1371/journal.pone.0246064

**Published:** 2021-01-29

**Authors:** Phillip M. Jolly, Hubert Van Hoof, Feier Chen, Bora Kim, Mateo Estrella Duran, Ana Cueva Navas, Julio Gavilanes Valle, Gabriela Maldonado Pérez

**Affiliations:** 1 School of Hospitality Management, College of Health and Human Development, Pennsylvania State University, University Park, Pennsylvania, United States of America; 2 School of Hospitality and Tourism Management, University of Surrey, Guildford, England, United Kingdom; 3 Universidad de Cuenca, Cuenca, Azuay, Ecuador; 4 Universidad Central del Ecuador, Quito, Pichincha, Ecuador; 5 Escuela Superior Politecnica del Litoral, Guayaquil, Guayas, Ecuador; 6 Pontificia Universidad Catolica del Ecuador, Quito, Pichincha, Ecuador; University of Georgia, UNITED STATES

## Abstract

Cultural tightness-looseness represents the degree to which a particular culture possesses strong behavioral norms, and the degree to which members of that culture are likely to sanction individuals who deviate from those norms. While tightness-looseness has been quantified for a large and growing number of countries around the world, there are many countries where a tightness-looseness score has yet to be determined, thus impeding the inclusion of those countries in cross-cultural research with a tightness-looseness focus. There is a dearth of research on cultural tightness-looseness in South America in particular. We report results from a national survey of 1,265 Ecuadorian residents which provided quantification of the relatively tight culture of Ecuador.

## Introduction

Cross-cultural psychology has provided important insights into the ways that cultures can influence affect, cognition, and behavior, and how psychological phenomena may differ depending on the contextual cultural factors that surround individuals. While a significant body of research has been developed around many cultural differences, researchers have paid significantly less attention to cultural issues in some parts of the world as compared to others. Latin America in particular has been understudied by scholars seeking to quantify cultural characteristics at the country level, and many Latin American countries can therefore not be included in country-level comparative research. The present study is focused on broadening our understanding of cross-cultural psychology by providing insight into the important construct of cultural tightness-looseness in the Latin American country of Ecuador.

Cultural tightness-looseness (CTL) [1] is a robust and stable characteristic of a culture [2] which refers to the degree to which cultures either have strong social norms and a low tolerance of deviant behavior (tight cultures) or weak social norms and a high tolerance of deviant behavior (loose cultures). Existing research has tied CTL to a range of important individual-, regional-, and national-level outcomes such as entrepreneurship and new venture creation [3], creativity [4], well-being [5], reactions to fraud [6], and perceptions of leadership [7]. In addition, CTL has been identified as a promising theoretical framework for the investigation of a number of phenomena in consumer behavior [8], hospitality and tourism [9], and religious and cultural diversity [10], to name but a few fields.

Importantly, many of these studies use existing, secondary or archival measurements of CTL from previous studies [e.g. 11, 12]. That is, many researchers operationalize CTL using pre-existing CTL scores taken from seminal CTL work [1, 13], rather than collect this as primary data in their own data collection procedures. This has led to calls to broaden the availability of CTL data at both the national and regional levels [1], as providing CTL scores for additional countries will facilitate greater cross-cultural and within-country research on the effects of CTL. While CTL has been quantified for hundreds of cultures/countries around the world [1, 13], all 50 US states [14], and the 31 Chinese provinces [15], surprisingly little CTL research has focused on Latin America. This gap is a glaring one, given that cross-cultural research helps to establish an understanding of cultural differences that can improve communication and collaboration around the world [16].

This brief report seeks to help narrow this gap, by reporting on CTL at the national level for the country of Ecuador. As part of a larger research project, the present study collected 1,265 survey responses across the country which were used to calculate a CTL score for Ecuador. Our findings contribute to cross-cultural psychology research in several ways. First, we provide evidence of the validity of the most widely-used CTL measure and its factor structure in a novel population. Second, and perhaps more importantly, we report information on a country-level CTL score for Ecuador, one of the largest countries by population that has, to date, not been included in any country-level cross-cultural research focusing on CTL. This information will hopefully facilitate the inclusion of Ecuador in a greater number of future cross-cultural studies focused on CTL.

### Introduction to Ecuador

Ecuador is one of the smallest and most diverse countries in South America. Located on the Equator in the north-western quadrant of the continent, the country’s 95,000 square miles are divided into four regions, each with its own distinct climate, economic, topographical, socio-cultural and demographic characteristics. The continental regions, which run the length of the country and are separated by the Andes mountain range, are the coastal region (“La Costa”), the mountain region (“La Sierra”), and the eastern or Amazon region (“El Oriente”). The Galapagos Islands, Ecuador’s fourth region, are located some 800 miles off the coast in the Pacific Ocean.

The country’s population of roughly 17.5 million inhabitants is comprised mainly of Mestizo (79.0%), Indigenous (7.0%), Amerindian (7.0%), Caucasian (6%) and Afro-Ecuadorian (3.0%) population groups. About two-thirds of the population is urban-based and centered around the country’s three largest cities, Guayaquil, Quito and Cuenca. Approximately 75% of the population is Catholic. The country’s annual population growth rate has ranged from 1.5% to 2.0% per year over the past twenty years. The average age is a relatively young 28 years (in neighboring countries such as Brazil, Argentina, Colombia, Peru and Chile this ranges from low-to mid-thirties) and the average life expectancy is 78 years of age [17].

Ecuador struggles with high poverty (25%), and high unemployment (8%) [18], an underdeveloped healthcare system, high risk of infectious disease, a struggling and underfunded education system, poor public infrastructure, and mounting corruption at all levels of society. In addition, the country is prone to natural disasters such as earthquakes, flooding, and volcano eruptions and has been involved in a series of territorial conflicts that date to the Spanish conquest of the Inca Empire in 1533 [17]. Its average net annual salary hovers around $35,000 and its monthly minimum wage was $424 in 2019. Traditionally, the main sources of revenue have been oil (about 35% of GDP) and agricultural products (such as shrimp and bananas); together the top three export items total approximately USD 13 billion [19]. Data shows that the national economy deeply relies on the exportation of natural resources with limited industrialization, which is similar to other nations of the region [20].

### Cultural tightness-looseness in Ecuador

Tightness-looseness of a culture has been theorized to be related to a range of ecological and societal threats as well as reflected in the importance of certain social institutions and customs, given that such threats necessitate strong social coordination to ensure survival of a particular population [1]. Conceptually, cultures that developed under the pressure from significant social, political, and natural threats focused on enforcing strong behavioral norms and punishing deviance from those norms, because these norms enhanced the social coordination that was necessary to ensure survival [1]. Given Ecuador’s history of territorial conflict, prevalence of communicable diseases, relative resource scarcity, and proneness to natural disasters, we expected the culture of Ecuador to be relatively tight (strong social norms and low tolerance for deviant behavior), which was defined in this study as being above the midpoint of the CTL measure. This expectation is further supported by the high level of religiosity in Ecuador, as well as significant government influence over media, which have been found to reflect the tightness-looseness of a culture [1].

## Methods

Four Ecuadorian collaborators, representing four of the country’s leading universities, oversaw data collection via trained students who collected data in the field. In an effort to reach as many different regions as possible, students from the Universidad Central and the Universidad Católica collected data in Quito and in the Esmeraldas, the northern part of the coastal region. Students from the Universidad de Cuenca collected data in Cuenca in the heart of the mountain region, and students from ESPOL (Escuela Superior Politécnica del Litoral) collected data in Guayaquil and the central and southern parts of the coastal region. Due to lack of accessibility, data were not collected in the Galapagos and Amazon regions.

The research protocol was approved by the University of Cuenca prior to data collection and all data transmitted to the United States was fully anonymous. Oral consent for participation was obtained by the students who collected the data. All potential participants were informed that the data were anonymous and that no identifying information would be collected. Participants were also instructed that they were free to not answer any questions and that they should not write their name or any other identifying information on the survey. Surveys were administered, collected, and processed by the Ecuadorian authors. Only anonymous data were shared with authors in the United States, therefore Penn State researchers were not involved in human subjects research as defined by the US Department of Health and Human Services, and IRB approval at PSU was not necessary.

The data was collected over a period of three months, from late July till November 2019. In order to collect data, trained students approached individuals in their personal networks (fellow students, coworkers, family members, church members, etc.) and approached random people in public spaces (bus stops, markets, etc.) to ask for their participation. After responses were collected, they were identified by region and city, and the surveys were sent to a central data processing location at the University of Cuenca, where a team of trained students input the survey responses into a spreadsheet. Subsets of data entry were cross-checked by one of the authors who supervised data entry to guard against data-entry mistakes. The master spreadsheet was divided by region of origin and by questions asked and then sent to collaborators in the United States for analysis.

### Measures

In order to quantify CTL, we employed the six-item perceived cultural tightness-looseness measure developed by Gelfand and colleagues [1] measured on a 1–6 scale from “strongly disagree” to “strongly agree.” Prior to survey administration, the survey was translated into Spanish by two co-authors who are fluent in both English and Spanish and then back-translated into English by another co-author who is also fluent in both languages. This translation-back translation procedure ensures that the items retain their original meaning when translated from English into Spanish [21]. In addition to CTL, the survey also collected a range of demographic measures, such as age, gender, student status, employment status, work experience, educational attainment, income level, and religious affiliation.

### Sample characteristics

The final sample consisted of 1,306 responses. After dropping responses that included missing data on the CTL measure, our analysis sample consisted of 1,265 responses spread across two geographical regions of Ecuador. The average age of respondents was 23.81 years (sd = 12.94 years). Our sample was 52% female and 39.4% were university students. Approximately 61.5% of the respondents were employed and reported an average of 11.15 years (sd = 11.49 years) of work experience. The distribution of highest level of educational attainment in our sample was 4.8% primary school, 33.0% secondary school, 54.0% tertiary education, and 8.3% graduate or professional school. Monthly income was reported in US Dollars in ranges of no income (4.7%), up to $500 (47.6%), $501–$1,000 (28.1%), $1,001–$1,500 (11.5%), $1,501–$2,000 (3.1%), and $2,001 + (5.0%). Finally, 65.9% of respondents were Catholic, 6.1% were Protestant, 6.5% were atheist, and 21.5% reported a religion of “other.”

### Analysis and results

To establish the fit of our measurement model for CTL we ran a confirmatory factor analysis using robust diagonally-weighted least squares estimation using the *lavaan* package in R, because this estimation technique is better at controlling for Type I error when assessing model fit with ordinal indicators [22]. The six-item, single-factor model (χ^2^ (9) = 271.51, p < .001, CFI = .95, RMSEA = .12, SRMR = .07) demonstrated an acceptable to good fit to the data [23]. We report RMSEA because it is the most commonly reported index of fit in structural equation modeling. However, we note that because the individual items of the CTL measured are on an ordinal scale, RMSEA is biased toward rejection in large samples, SRMR is a more appropriate fit statistic, and the reported results for RMSEA should not be taken to indicate a lack of model fit [24]. Furthermore, because item-level data were ordinal, we calculated ordinal alpha [25], which indicated that the measure had an acceptable internal consistency (α = .71). We averaged the mean scale score for CTL across all respondents, in order to establish an overall country-level score for Ecuador [1]. This analysis indicated that the CTL score for Ecuador is 4.00 (sd = 0.84). This value, which indicates a relatively tight culture in Ecuador, is in line with previously reported scores for other South American countries, such as 3.7 for Venezuela and 3.5 for Brazil [1]. Finally, in order to explore the relative tightness or looseness of Ecuador, we used a one-sample t-test to determine whether the CTL score for Ecuador (4.00) was significantly different from the midpoint of the measurement scale (3.5). Results (*t*(1264) = 21.15, p < .001) indicated that the score for Ecuador is significantly different from the midpoint of the scale. Because higher scores on the CTL measure indicate tighter cultures, this provides evidence of a relatively tight culture in Ecuador.

### Additional analyses—Group differences

In order to determine whether there were differences in CTL responses based on demographic or geographical variables, we conducted an exploratory analysis of covariance (ANCOVA) to determine whether CTL scores varied as a function of participant age, gender, student status, employment status, work experience, marital status, educational attainment, income, religion, or region. As these were exploratory analyses, we had no a priori hypotheses with respect to possible effects of demographic characteristics. Only one grouping variable, region, exhibited significant differences in CTL scores. A pairwise comparison with Bonferroni correction indicated that the coastal region (m = 4.12) exhibited a higher average CTL score when compared to the mountain region (m = 3.93, p < .001). While this difference is statistically significant, it is also fairly small in magnitude. An exploration of this regional difference is beyond the scope of this study, however it might be attributable to the composition of the regional populations or differences in the distribution of income and/or educational attainment between regions. This speculation would be in line with previous findings indicating that individuals of lower social class are likely to perceive the same culture as “tighter” than individuals from higher social classes [26] and other work explaining intra-country regional differences [14, 15]. We encourage further work that is designed to further investigate regional differences in CTL in Ecuador.

## Discussion

As evidenced by the large and growing number of citations to the seminal paper in the field [1], CTL is a widely studied and important dimension of cultural difference. However, an inability to quantify country-level cultural differences for many nations has meant these countries are left understudied. We believe that it is important to provide broad country-level scores on important cross-cultural variables so that future research may enhance its scope to include countries and regions of the world that have been traditionally overlooked [cf. 27].

The present study contributes to research in several ways. First, we provide evidence of the validity of the Gelfand et al. measure of CTL and its factor structure in a novel population. Second, the primary contribution comes through providing information on a country-level CTL score for Ecuador, a country of 17.5 million people that has, to date, not been included in any country-level cross-cultural research focusing on CTL.

Providing a country-level score will allow for studies that have integrated archival CTL data with other existing archival data that does include Ecuador to include Ecuador in their research. For example, Aktas et al. [11] used existing CTL scores from Gelfand et al. [1] and integrated them with existing data on perceptions of effective leadership from the Global Leadership and Organizational Behavior Effectiveness (GLOBE) project [28]. While the GLOBE data do include Ecuador, we make an assumption that the lack of existing CTL scores for Ecuador led to the country’s exclusion from the Aktas et al. study. Our study contributes to cross-cultural research by providing a tool necessary for Ecuador to be included in a larger range of future work.

### Limitations

As with any study, there are some limitations to our work that should be noted. First, many of our respondents were recruited through convenience sampling via the networks of the trained student personnel. Thus, our sample is made up of more highly-educated and higher-income individuals than it would be if the sample were truly representative of the Ecuadorian population; thus our findings should be interpreted in light of findings demonstrating that social class and economic insecurity may influence perceptions of CTL [26]. Furthermore, while we collected data in multiple regions of Ecuador, our samples were collected in only 3 of the country’s 24 provinces. While our data collection locations represent major population centers in different regions of the country, there may be differences at the level of intra-country administrative divisions such as provinces or states [cf. 14, 15]. Thus, future research should expand data collection to all provinces of Ecuador to determine where and to what degree intra-country differences in CTL may exist.

## Conclusion

While the current study presents a small step toward incorporating traditionally underrepresented and understudied nations into cross-cultural CTL research, we hope that our work sets a precedent for the reporting of more country-level CTL scores (and other national-level, cross-culturally relevant variables), as these findings can make important contributions to broadening the scope of cross-cultural research. Despite the increasing globalization of psychology [29], there remain significant gaps in our understanding of countries and cultures throughout the world. Only by providing the tools necessary for future researchers to investigate more countries and cultures will these gaps be narrowed.
